# A novel strategy for partial purification of alkane hydroxylase from *P. chrysogenum* SNP5 through reconstituting its native membrane into liposome

**DOI:** 10.1038/s41598-024-54074-0

**Published:** 2024-02-15

**Authors:** Satyapriy Das, Sangeeta Negi

**Affiliations:** Department of Biotechnology, Motilal Nehru National Institute of Technology Allahabad, Prayagraj, U.P. 211004 India

**Keywords:** Alkane hydroxylase (AlkB), Membrane reconstitution, Fluorescence microscopy, Atomic force microscopy, Particle size analysis, Biological techniques, Biotechnology, Microbiology, Structural biology

## Abstract

Integral proteins or enzymes are still challenging to purify into their native state because of their need for an amphipathic environment and cofactors. Alkane hydroxylase (AlkB) is a membrane-bound enzyme that catalyzes the hydroxylation of a range of alkanes that have a broad spectrum of applications. In the current study, a novel approach has been explored for partial purification of alkane hydroxylase (AlkB) in its native state through restructuring the lipid bilayer of *Penicillium* *chrysogenum* SNP5 into a liposome to extend the native and protective environment to AlkB enzyme. Three different methods i.e., reverse-phase evaporation method (RPEM), detergent-based method (DBM), and ethanol injection method (EIM) have been used for reconstituting its native membrane into liposome. On characterizing liposomes through fluorescence imaging, AFM, and particle size analysis, the reverse-phase evaporation method gave the best results based on the size distribution (i.e., 100–300 nm), the morphology of liposomes, and maximum AlkB specific activity (i.e., 140.68 U/mg). The maximum reconstitution efficiency of 29.48% was observed in RPEM followed by 17.3% in DBM and 12.3% in EIM. On the characterization of the purified AlkB, the molecular weight was measured of 44.6 KDa and the thermostability of liposomes synthesized with the RPEM method was obtained maximum at 55 °C. This approach may open a new strategy for the purification of integral enzymes/proteins in their native state in the field of protein purification and its applications in diversified industries.

## Introduction

Alkane hydroxylase (AlkB) is a membrane-embedded integral protein that is a member of the oxidoreductase enzyme class. In recent years, AlkB has drawn a lot of attention for its tremendous application in the biotransformation and biodegradation of alkanes, particularly those in the C_5_–C_18_ range. By adding molecular oxygen, it catalyzes the hydroxylation of a variety of alkanes, turning them into the corresponding alcohols, aldehydes, carboxylic, epoxides, etc. forms in a sequential way^1^. Bacterial AlkB is well characterized as a nonheme integral protein comprised of three subunits: Alkane hydroxylase, Rubredoxin, and Rubredoxin reductase, a flavoprotein (AlkT)^2^. It has six transmembrane alpha helices, two short alpha helices partially embedded in the membrane, and another 7-membrane associated alpha helices outside the membrane, according to a recent discovery by Chai et al*.* 2023^3^. It is challenging to isolate AlkB in its native and functional state for industrial applications due to its intricate assembly in the lipid bilayer. Another aspect is its dependency on cofactors like NADH, nicotinamide, and Iron. In this line, a novel and unique strategic approach must be adopted where it can be isolated along with its cascade and cofactors.

Integral proteins like AlkB need a certain amphipathic microenvironment, hence, enclosing it in its own membrane along with its coenzymes after discarding intracellular components may develop the desired microenvironment and also can reduce downstream processing steps. The existing approaches for the purification of membrane-bound proteins, including detergents^4^, solvent systems^5^, protein-based nanodiscs^6,7^, and styrene-maleic acid copolymers (SMALP)^8^, imitate lipid bilayers and offer an appropriate amphipathic environment to membrane-bound enzymes. Zhang et al*.* 2021 synthesized circularized nanodiscs (cNDs) using SpyCatcher-SpyTag technology to reconstitute membrane lipid bilayer for the study of chemical reactions/intermediates during synaptic transmission and viral entry and Ren et al*.* 2022 engineered nanodiscs as a sensor to study membrane signal transduction^9,10^. However, these approaches have limited application in the study of membrane functioning due to their complexity and high cost.

In order to extract and purify the membrane proteins various detergents are also used, which are readily available and inexpensive, but their severe solubilization conditions frequently cause disruption of protein structure and function. These drawbacks of detergents associated with the extraction and purification of membrane proteins restrict this technique from providing the desired physiochemical and amphipathic environment for membrane proteins. The Peptidisc technique is another approach for the purification of membrane proteins in their original structure and functionality. However, their uses become limited due to low commercial availability, lower stability, and high cost, hence they are still in their infancy and need to be further optimized prior to being brought into practice^11^. Reconstructing plasma membranes into liposomes has attained the interest of scientists for the purification of membrane proteins, which provide biophysical platforms to retain membrane proteins into their native confirmation by preventing denaturation, aggregation, and loss of protein function which has extensive applications in drug delivery, protein characterization, the study of the cellular processes, etc.^12,13^. It has been also investigated that the liposome provides excellent substrate permeability, substrate selectivity, stability, and thermostability to understand the various characteristics of complex membrane-bound proteins^14,15^.

Various other approaches like detergent-free isolation, reconstitution of membrane proteins using copolymers, solvents, commercial phospholipids for isolation of Cyt P450, NDH-2a enzyme, rhomboid protease GlpG, and many antibodies^16,17^ have been investigated. However, the activity of enzymes or proteins could not attain an acceptable level because of these methods due to the lack of required natural amphipathic conditions. However, these problems can be overcome by modifying the process of liposome synthesis, changing the process parameters, and reconstitution of membrane proteins into their own native lipid. Neves et al*.* 2009 reported the self-assembling of OmpF in DMPC on reconstitution of membrane proteins of *E.* *coli* into liposomes and Knol et al*.* 1998 reconstituted membrane of *Streptococcus* *thermophilus* to enclose lactose transport protein (LacS) by using a detergent-based method^18,19^. A giant unilamellar vesicle (GUV) was constituted through diverse synthesis methods on reconstitution of many membrane proteins^20^. Researchers observed that reconstitution of the cell membrane to enclose integral proteins is a very economical and efficient approach.

While preparing liposomes process parameters of reconstructing membranes play a critical role because pore size, structure, size of liposomes, microenvironment, the substrate–enzyme interaction, and mass transfer depend on it. For the synthesis of liposomes, reverse-phase evaporation, detergent-based, ethanol injection, or freeze-drying methods are used in general. All these techniques have different benefits and drawbacks; thus, the best technology must be chosen based on the needs of the protein being reconstituted. For example, detergent-based and reverse-phase evaporation approaches depend on the elimination of the detergent by dialysis and the organic solvent by evaporation, respectively. In these methods, sometimes detergents are used to solubilize the phospholipid layer and organic solvents lead to the denaturation of 50–70% of delicate enzymes^21^. Other techniques like lipid hydration and sonication may also denature the enzyme during the sonication process. To overcome such shortfalls Walde et al. 2001 used a freeze–thaw method for conversion of multilamellar liposomes to unilamellar liposomes, however, only 1–2% efficiency was achieved^22^.

In this study, a very innovative approach has been developed to purify AlkB from *Penicillium* *chrysogenum* SNP5 by encapsulating it in its own native cell membrane instead of using other phospholipids. To encapsulate AlkB, reverse-phase evaporation, detergent-based, and ethanol injection methods were used with slight modifications to prepare liposomes, and efficiency was compared on the basis of specific activity and thermal stability. Liposomes synthesized through all three methods were characterized through fluorescence microscopy, atomic force microscopy, and particle size analysis. Current findings would be helpful to purify and concentrate AlkB for its commercial applications in a broad spectrum.

## Material and methods

### Microorganisms and materials

*Penicillium* *chrysogenum* SNP5 (MTCC13144) strain was locally isolated from grease-contaminated soil of the diesel loco shed and identified by Microbial Type Culture Collection and gene bank Chandigarh, India^23^. Triton X-100, Phenylmethylsulfonyl fluoride (PMSF), Lauryl dimethylamine oxide (LDAO), Nicotinamide adenine dinucleotide (NAD) + hydrogen (NADH), 1, 6- diphenyl- 1, 3, 5- hexatriene (DPH), Acrylamide, Bis acrylamide, Coomassie brilliant blue R-250, Glycine, Bromophenol blue, Methanol, para-nitrophenol, 1-Bromooctane, Dimethyl Sulphoxide (DMSO), Petroleum ether, diethyl ether, other medium components, and Czapek-dox medium were procured from Sisco Research Laboratories Pvt. Ltd. (Mumbai, India). Hexadecane and THB (Tetrahydrobiopterin), Acetic acid, and Digitonin were procured from TCI Chemicals Pvt. Ltd. (India). Phosphatidylcholine and cholesterol were purchased from Sigma-Aldrich Chemicals Pvt. Ltd. PageRuler™ Plus Prestained Protein Ladder was procured from Thermo Scientific.

### Production of AlkB through submerged fermentation

Submerged fermentation was carried out in 250 ml Erlenmeyer flask with 100 ml of Czapek-dox broth (NaNO_3_-2.5 g/l, K_2_HPO_4_-1.0 g/l, MgSO_4_.7H_2_O-0.5 g/l, KCl-0.5 g/l, FeSO_4_-0.45 g/l), 1% hexadecane, 0.5% YEPD, 0.1% glucose and 1 mM TBH. The flasks were autoclaved at 121 °C, 15psi for 15 min. 1.0 ml spore suspension of *Penicillium* *chrysogenum* SNP5 (with spore concentration of 1.4 × 10^7^ spores/ml) was added to each flask and incubated at 28 °C for 12 days and growth was observed.

### Cell lysis and preparation of membrane lysate

Cells were harvested by centrifuging it at 7826 g for 10 min at 4 °C, and the pellet was washed 3 times with Tris–HCl buffer. Preparation of membrane lysate is the first and crucial step towards the synthesis of liposomes which results in the removal of intracellular materials and to separate the membrane fragments. To prepare the membrane lysate, cells were sonicated with an ultrasonic cell crusher (Model SKL-500D) Ningbo Haishu Sklon Electronics Instrument Co, Ltd. (Mainland, China) by taking 5 g of cell pellets and adding 3 times the volume of lysis buffer (150 mM NaCl, 20% (v/v) glycerol, 50 mM Tris HCl, 1 mM Digitonin, 2% Triton X-100 and 1 mM PMSF) at 50 kHz using a 5 s on, 5 s off pulsating cycle for 5 min and centrifuged at 7826 g for 10 min to remove the intracellular materials.

### Liposome synthesis and reconstitution of membrane lysate of Penicillium chrysogenum SNP5

The liposome synthesis or reconstitution of the membrane with AlkB was simultaneously performed by further sonicating the first pellet at 70 kHz using a 9 s on, 9 s off pulsating cycle for 5 min in appropriate solvents/ buffer to synthesize the liposome where three different methods such as ethanol injection method^24^**,** detergent-based method^25^**,** and reverse-phase evaporation method^26^ were used with slight modification in existing protocols (cell membrane fraction was taken instead of phospholipid into solvent/detergent) along with a control liposome prepared by phosphatidylcholine and cholesterol in 3.57:1 molar ratio as per protocol described by Shu et al. (2019)^27^.

#### Reverse phase evaporation method (RPEM)

In the reverse phase evaporation method, 1gm of cell pellet was taken and suspended in 10 ml of chloroform/methanol mixture (2:1) and further sonicated at 70 kHz using a 9 s on, 9 s off pulsating cycle for 5 min to solubilize the membrane lipid into chloroform/methanol mixture. After the proper solubilization, the solvents were evaporated at 45–60 °C with continuous stirring and the obtained dry film was redissolved in the aqueous phase (Tris–HCl buffer) with continuous stirring for 1–2 h to promote the reconstitution of membrane proteins into liposome^26^.

#### Detergent-based method (DBM)

The detergent-based method used for liposome synthesis by taking a 1gm cell pellet which was lysed in the above-mentioned detergent containing lysis buffer at 70 kHz using a 9 s on, 9 s off pulsating cycle for 5 min to solubilize the membrane lipid into a mixture of digitonin and triton X-100. After solubilization, the detergent and membrane lipid solution were hydrated and then diluted with Tris buffer which results in the spontaneous formation of micelle due to a decrease in detergent concentration. After that, the detergents were removed by the repetition of dialysis of the solution against Tris HCl buffer^28^.

#### Ethanol injection method (EIM)

The reconstitution of AlkB with ethanol injection method was done by taking 1gm of cell pellets in 10 ml of ethanol and sonicated for and then sonicated at 70 kHz using a 9 s on, 9 s off pulsating cycle for 5 min to solubilize the membrane lipid into ethanol. The solubilized membrane lipid was then loaded in a sterile syringe and injected into preheated Tris HCl buffer at continuous stirring. The dilution of the membrane lipid-ethanol mixture in aqueous buffer resulted in the reconstitution of AlkB in membrane lipid as vesicle form finally entire ethanol was evaporated and the aqueous phase containing liposome was preserved for further experiments^29^.

### Fluorescence staining and imaging of liposome

Fluorescence imaging of liposomes was done with a fluorescence microscope (model BX51) by Olympus (Japan) using DPH dye. The dye was used at 100 μM concentration and incubated for 1 h with unprepared and prepared liposomes and with control for each method. After incubation, slides were made for each of the samples and analyzed under a fluorescence microscope at 20 μM scale^30^.

### Atomic force microscopy (AFM) of liposome

For 3D visualization of synthesized liposomes (reconstructed membrane), AFM was performed by Agilent (5500). Samples of liposomes were prepared by spreading liposome suspension over a glass slide (1 × 1 cm^2^) with the help of a spin coater and dried from the air (65% humidity) for two hours. All the images were obtained with non-contact mode using a triangular cantilever which was 100 nm long and had a spring constant of 0.03 N/m. The characterization was performed with a scan angle of 90° and a 70% approach^31^.

### Particle size analysis of liposome

The particle size of the liposome was measured at a constant temperature (25 °C ± 2°) using Microtrac, Inc. (Montgomeryville PA 18,936 USA) which uses a 52-detector array. The pump speed, stirrer speed, and ultrasonic level were set at 2000 rpm, 800 rpm, and 100% respectively. Particle size was calculated on a volume basis using the Mie theory and Malvern proprietary software FLEX (version 11.0.0.4). Analysis settings used were the General-Purpose Model with the Irregular Shape Mode. The particle refractive index and particle absorption index used were 1.52 and 0.01 respectively and were the same for both red and blue lasers. The refractive index of the Tris–Hcl buffer used as the dispersant was 1.34–1.35^32^.

### Alkane hydroxylase activity assay with NADH method in free enzyme and synthesized liposomes

Alkane hydroxylase (AlkB) activity of synthesized liposomes was measured by a continuous method with slight modification. The reaction mixture for the free enzyme assay contained 100 mM Tris HCl buffer (pH-7.4), 0.035% LDAO (1.5 CMC), 20% glycerol, 100 μl free enzymes, and 1 mM hexadecane. While measurement of the AlkB activity in all synthesized liposomes was performed in a reaction mixture of 100 mM Tris HCl buffer (pH-7.4), 20% glycerol, 100 μl liposomal enzyme, and 1 mM hexadecane. A mixture lacking NADH was used as a negative control. The reaction mixture was then incubated at room temperature for 20 min. The reaction was initiated by adding NADH to a final concentration of 50 mM. The rate of NADH consumption was determined by monitoring the change in absorbance at 340 nm at room temperature for 10 min. One unit is defined as the amount of enzyme required for the consumption of 1 mM of NADH (ε340 = 6220 M^−1^ cm^−1^) per minute^33^. The specific activity of AlkB was calculated by measuring the protein content present in reconstituted as well as free enzyme samples by the lowery method^34^.

### Alkane hydroxylase activity assay with 8-pnpane method in free enzyme and synthesized liposomes

**Figure 1:**
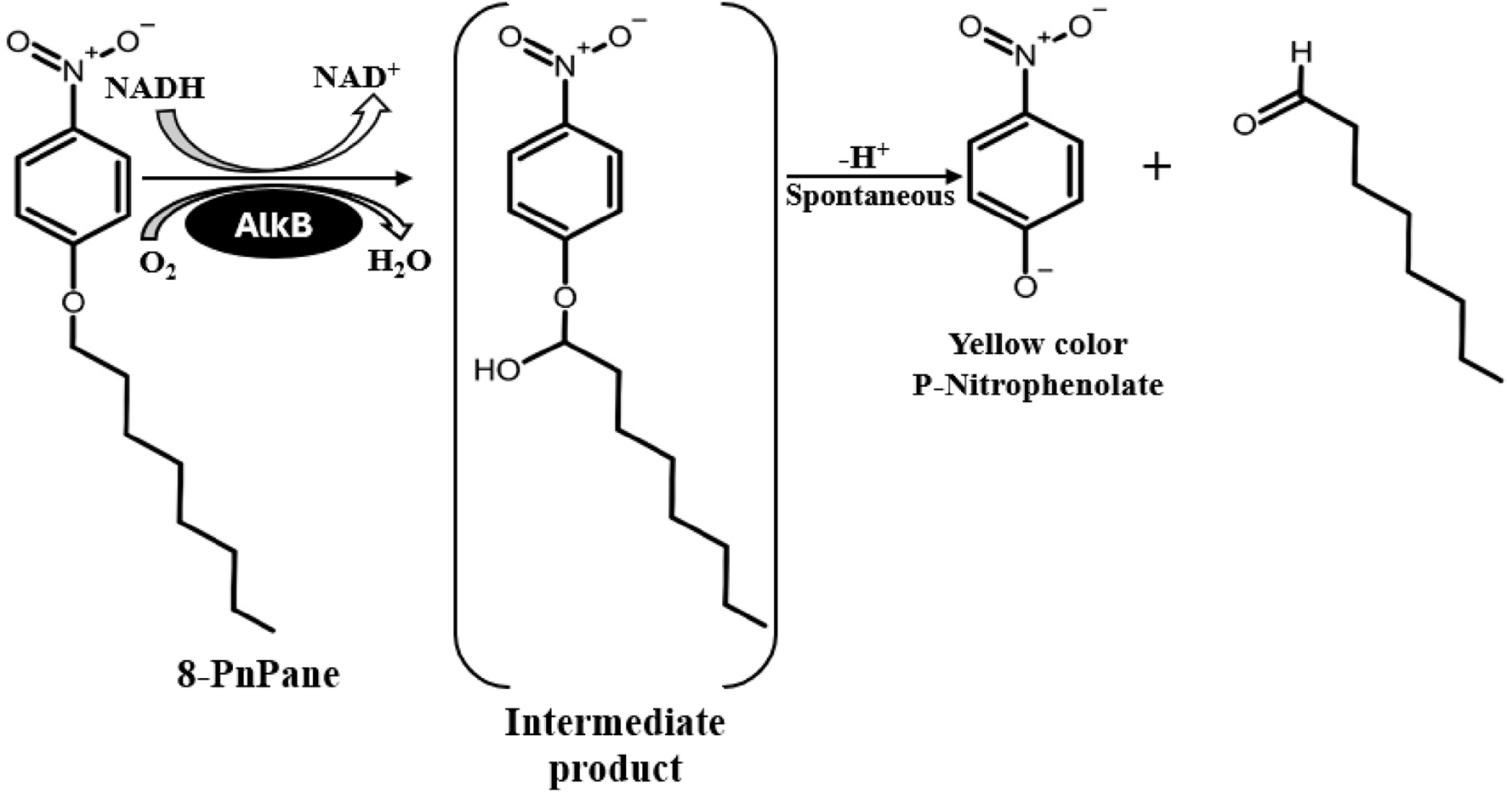
Working principle of 8-pnpane method used for enzyme assay and Zymography of AlkB.

The 8-pnpane is the surrogate substrate consisting of a C_8_ carbon backbone and a p-nitrophenyl moiety that is mostly used as colorimetric screening of P450 mutants. Its hydroxylation results in the generation of unstable hemiacetal yellow color p-nitrophenolate and aldehyde that can be easily read at 410 nm (Fig. 1)^35^ We used this concept for the assay of our liposome reconstituted AlkB. The 8-pnpane was synthesized as the method described by Farinas et al. 2001, where 5.18 mM 1-Bromooctane and 5.71 mM 4-nitrophenol were refluxed in 30 ml DMSO at 120 °C for 5 h. The DMSO was removed by distillation till the dryness. The obtained brown residue was purified with a silica column by eluting the sample in a mixture of petroleum ether and diethyl ether (10:1)^36^. The enzyme assay was performed in triplicate by taking 8-pnpane as substrate in 96 well plate microtiter plate. The reaction mixture consisted of 100 mM Tris HCl buffer (pH-7.4), 150 µM 8-pnpane in 1% DMSO, 100 µM of liposomal/free enzyme, 20% glycerol, and the reaction mixture was incubated at room temperature for 20 min and 1 mM NADPH was added and absorbance was taken at 410 nm in multimode ELISA plate reader.

### Native PAGE and Zymography of reconstituted AlkB

To confirm the reconstitution of cell membrane into liposome enclosing AlkB through all methods, zymography of AlkB was performed followed by Native-PAGE^37^. The 8-pnpane was used as colorimetric screening of the AlkB band in Native-PAGE gel. Similar chemistry of AlkB and 8-pnpane has been used for zymography as explained in previous methodology for the measurement of AlkB activity with 8-pnpane. This reaction mechanism underlying AlkB's activity with 8-propane on native PAGE gel has been explained in Fig. 1. To perform the Native-PAGE, AlkB/protein samples were prepared by lysing the liposome in Triton X-100, and protein samples were concentrated on dialysis against a 4 M sucrose solution. The native page and zymography were carried out by preparing separate polyacrylamide gels where the first gel consisted of 11% resolving gel and 4% stacking without SDS. While 2nd gel consisted of 11% polyacrylamide along with a zymography reaction mixture (100 Tris–HCl, 20% glycerol, 0.035% LDAO, 100 µl 8-pnpane and 50 µl of NADPH). Both gels were casted in a vertical minigel unit and allowed to polymerize. A 12 µl of each liposomal sample was loaded with loading dye lacking beta-mercaptoethanol and SDS in the first gel along with a protein marker (Thermo Scientific). The first gel was initially run for 30 min at 80Volts and then for 3 h at 105Volts, after that, the gel was disassembled and overlayed on the second gel having impregnated 8-pnpane and immersed in the enzymatic reaction mixture. After incubation first gel was stained with CBB-250 and destained to visualize the protein bands on the gel.

### Determination of reconstitution efficiency of liposome with AlkB

The % reconstitution efficiency of liposomes was determined by following the previously described protocol^38^ with slight modification. The liposome suspension was centrifuged at 9450 g for 2 h and the specific activity was measured in each pelleted liposome. The AlkB-specific activity was also measured in the supernatant of liposome after disruption with 1% Triton X 100. Reconstitution efficiencies were calculated in each liposome with the following formulae:

$$Reconstitution\; efficiency \left( {RE} \right) = \frac{Total \;AlkB \;specific\; activity - AlkB \;specifc\; activity\; in \;supernatent}{{Total \;AlkB \;specific\; activity}}$$

### Determination of thermostability of reconstituted AlkB

The thermal stability of reconstituted liposomes of AlkB was determined in each suspension according to^39^. The encapsulated enzymes and free enzymes were suspended in 100 mM Tris HCl buffer, pH 7.4, and incubated for 20 min in a water bath at varying temperatures, ranging from 20 to 60 °C. After the incubation liposome suspension was transferred into an ice bath and then checked for enzyme assay.

## Results

### Liposome synthesis and its fluorescence staining

**Figure 2:**
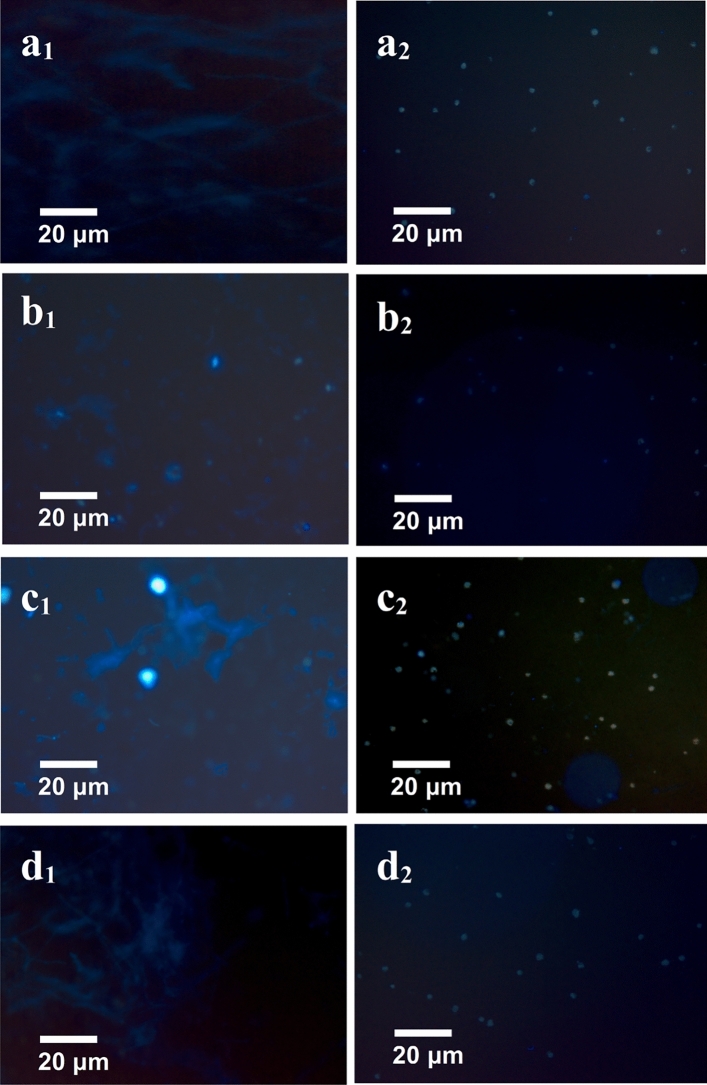
Fluorescence microscopy of DPH labeled liposome synthesized with three different methods along with control (i.e., a reverse-phase evaporation method, detergent-based method, and ethanol injection method respectively). (**a**_**1**_,**b**_**1**_,**c**_**1**_,**d**_**1**_) shows dissolved membrane lipid before liposome synthesis, and (**a**_**2**_**,b**_**2**_**,c**_**2**_**,d**_**2**_) show the synthesized liposome.

Liposome offers an amphipathic environment with lipid bilayer membranes and aqueous space in the form of a spherical vesicle to keep the enzymes/ protein highly stable in their native state^40^. In the present study, AlkB of *Penicillium* *chrysogenum* SNP5 was extracted first after cell lysis, and then the disrupted membrane was reconstructed. To achieve efficient entrapment of AlkB, different approaches of liposome synthesis have been tested and liposome synthesis was confirmed through fluorescence imaging by labeling with the DPH dye (Fig. 2). While exploring different protocols, the ethanol injection method shows the presence of very few numbers of liposomes and heterogeneous distribution after DPH staining (Fig. 2b_2_). The fluorescence images generated from DPH staining in REPM indicate that there is a proper synthesis of liposome (Fig. 2d_2_) and dissolved lipid before hydration (Fig. 2d_1_) when compared with control (Fig. 2a_1_,a_2_). In the case of the detergent-based method, the fluorescence image confirmed the synthesis of liposomes (Fig. 2c_2_) and dissolved lipids before dialysis or removal of detergent (Fig. 2c_1_) as compared to the control (Fig. 2a_1_,a_2_). However, there is a heterogeneous distribution of liposomes can be seen in the DBM method as compared to the control. When compared to other methods, it was observed that liposomes of homogeneous sizes were synthesized through the reverse-phase evaporation method (Fig. 2). The overall fluorescence images clearly indicate that RPEM method has proper synthesis of liposomes among all three methods (Fig. 1d_2_) as compared to the dissolved lipid before hydration (Fig. 2d_1_) and control (Fig. 2a_1_,a_2_).

### Atomic force microscopy of liposome

**Figure 3:**
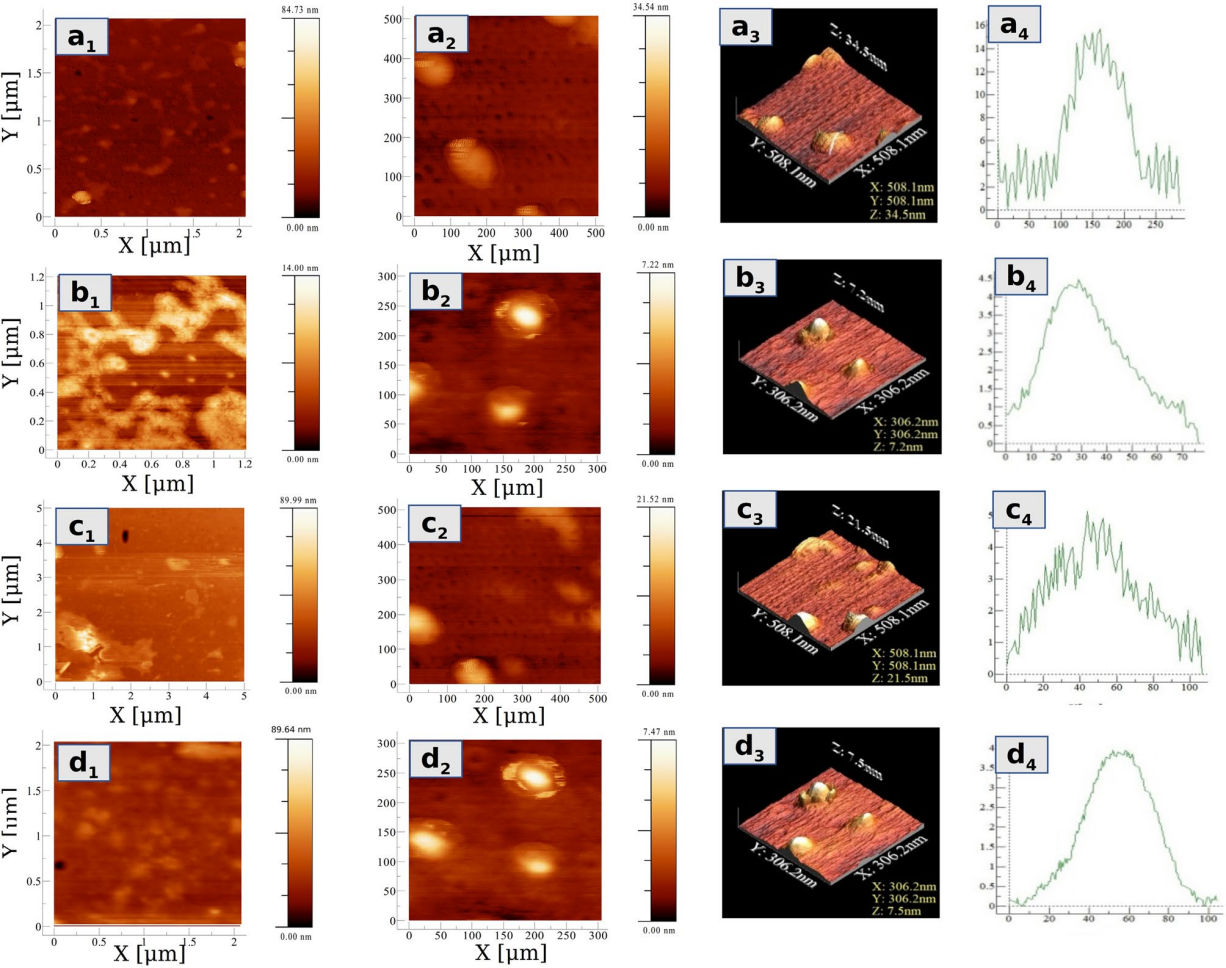
AFM images and height profiling of liposome synthesize through different methods along with control which was coated on glass substrate from aqueous suspension and dried for observation. (**a,b)** is the 2D images of liposomes at 5μm and 1μm scale, (**a**_**3**_**)** shows the 3D image, and (**a**_**4**_**)** is the vertical profiling of the 3D structure of liposome in control. Similarly, **(b**_**1**_**–b**_**4**_**)** represents EIM, **(C**_**1**_**–C**_**4**_**)** represents DBM and **(d**_**1**_**–d**_**4**_**)** represents REPM.

Atomic force microscopy offers direct imaging of the liposome morphology supported on a solid surface in aqueous media^31^. The synthesized liposomes were visualized by AFM and observed that the adherence of the spherical structure when it was scaled from the micrometer to nanometer range (Fig. 3). The height and diameters of the observed spherical structure were measured at 4–15 nm and 70–150 nm respectively. These results confirm that the spherical structures are liposomes as reported by^41^. Three different methods along with control (Fig. 3a_1_–a_4_) were compared by AFM imaging and well-shaped and uniformly distributed structures of liposomes were observed in the reverse-phase evaporation method (Fig. 3b_1_–b_4_). The detergent-based method poses variation in liposome size (Fig. 3c_1_–c_4_), as observed in fluorescence imaging whereas fewer numbers of liposomes could be seen in the ethanol injection method (Fig. 3d_1_–d_4_).

### Particle size analysis

**Figure 4:**
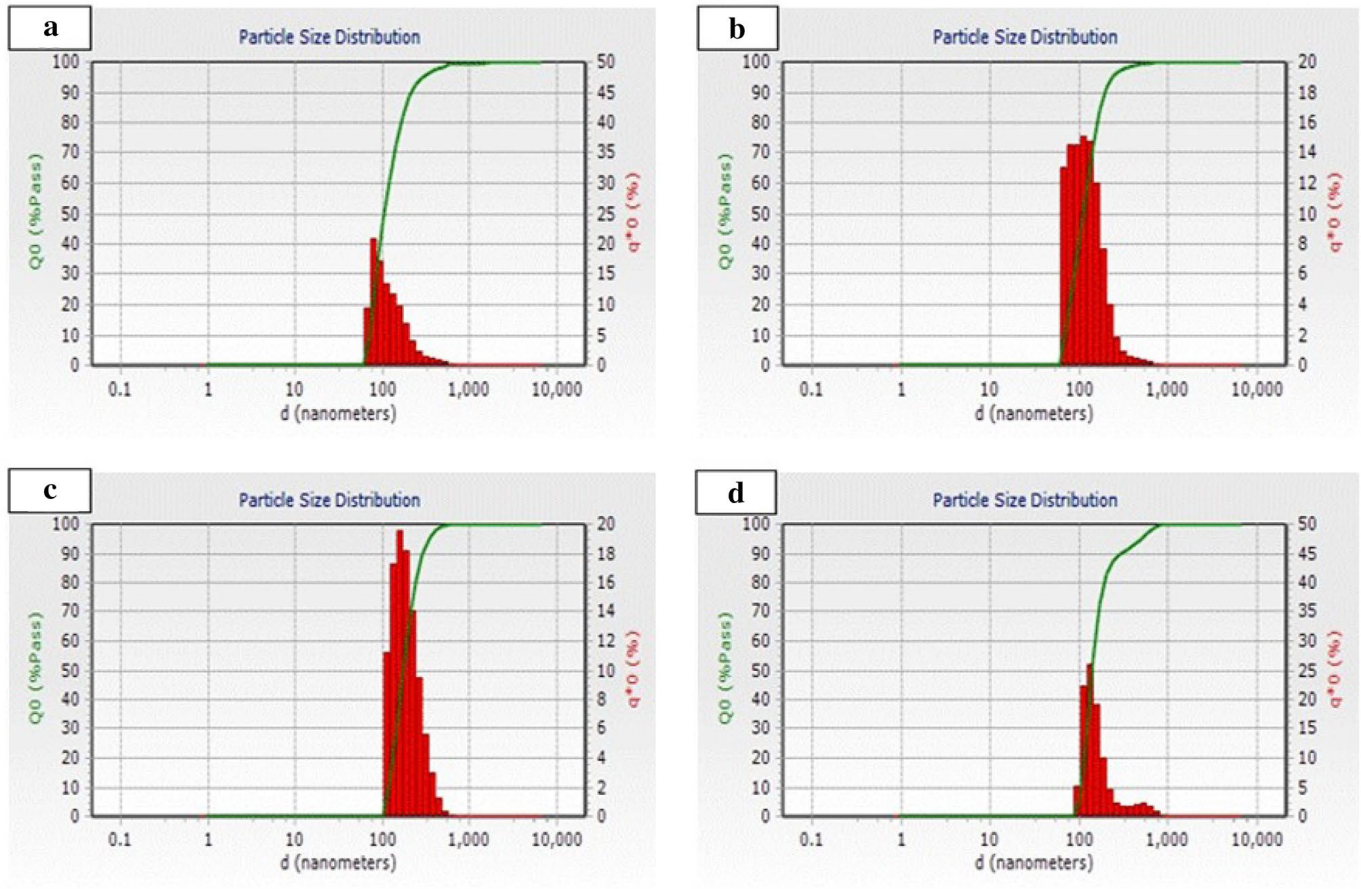
Size distribution of liposome particles suspended in Tris–HCl buffer. **(a)** Shows liposomes synthesized with phosphatidylcholine and cholesterol as a control. **(b)** Shows liposome distribution, synthesized from the reverse-phase evaporation method. **(c)** Shows the liposome distribution synthesize from the detergent-based method**. (d)** Shows the liposome distribution synthesize from the injection method.

The diameter and population of liposomes were measured through a particle size analyzer along with control (phosphatidylcholine and cholesterol) employing the dynamic light scattering method. The major populations of the liposome range from 100 to 300 nm in all the methods as shown in Fig. 4. The reverse-phase evaporation method shows particle distribution from 100 to 700 nm, but the major liposome population ranged from 100 to 300 nm (Fig. 4b), The detergent-based method also renders a size range of 100–600 nm, but most of the populations have an irregular distribution of liposomes (Fig. 4c). In the ethanol injection method, particle size distribution falls into the range of 100–300 nm but there is bimodal size distribution can be observed in liposome produced by EIM method (Fig. 3d).

### Measurement of AlkB-specific activity

**Figure 5:**
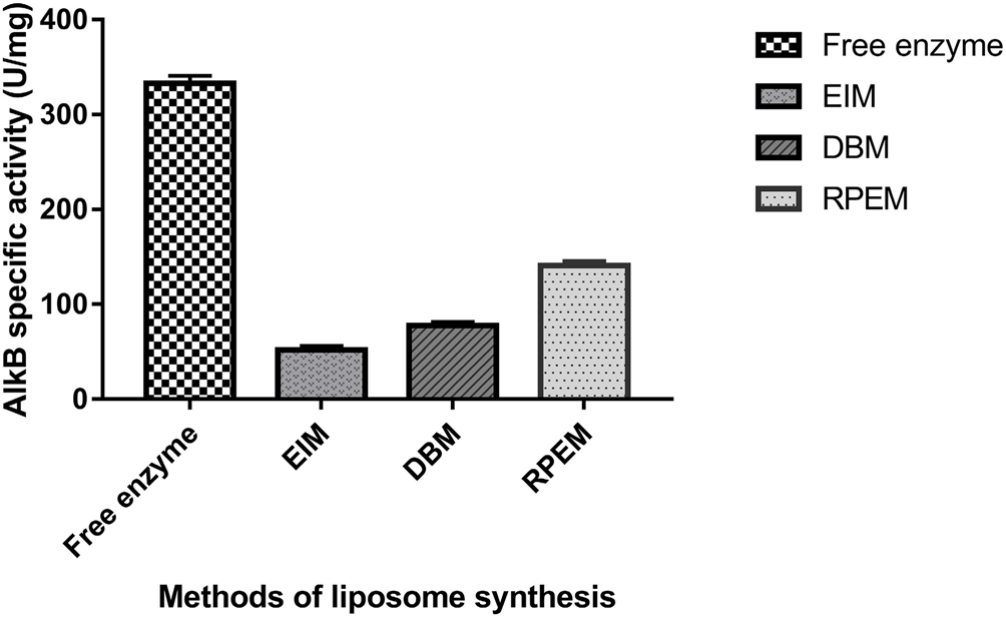
Specific activity of reconstituted AlkB from different methods. Data are shown as the mean of triplicate data after a one-way ANOVA test which indicates statistical significance (*P* < 0.001* and n* = 3).

**Figure 6:**
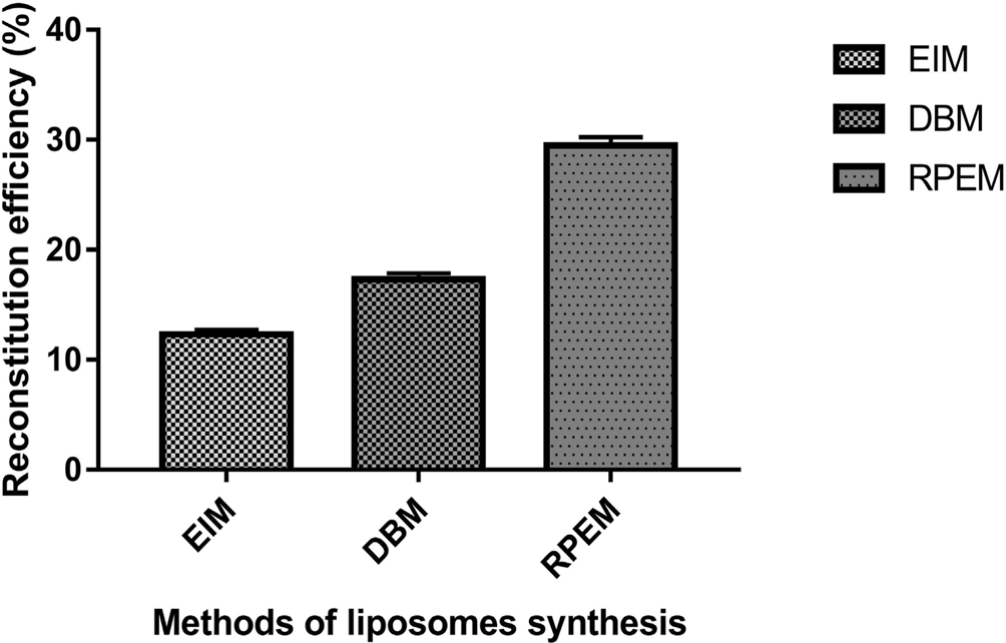
Reconstitution efficiencies of different methods of liposome synthesis. Data are shown as the mean of triplicate data after a one-way ANOVA test which indicates statistical significance (*P* < 0.001* and n* = 3).

The encapsulated AlkB was subjected to the enzyme assay and maximum specific activity was found in the reverse-phase evaporation method (140.68 U/mg) followed by the detergent-based method (77.46 U/mg) and ethanol injection method (51.79 U/mg) (Fig. 5). Similarly, the maximum reconstitution efficiency of 29.48% was observed in RPEM followed by 17.3% in DBM and 12.3% in EIM (Fig. 6).

### Alkane hydroxylase activity assay with 8-pnpane method in free enzyme and synthesized liposomes

**Figure 7:**
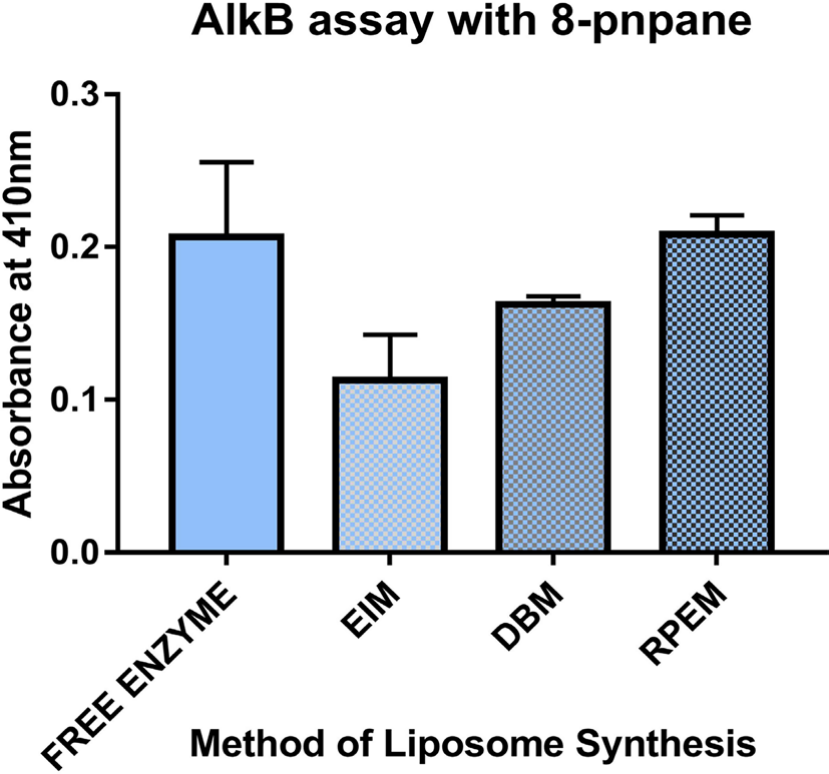
AlkB assay with 8-pnpane in different reconstituted liposomes. Data are shown as the mean of triplicate data after a one-way ANOVA test which indicates statistical significance (*P* = 0.0125* and n* = 3).

8-pnpane is the specific substrate to alkane hydroxylase which confirms the presence of the AlkB enzyme in a cell lysate or in any immobilized matrix. The enzyme assay with all liposome was carried out by incubating the enzymes with 8-pnpane and found that there is the highest absorbance with the RPEM method followed by DBM and EIM when compared with free enzyme (Fig. 7). This results complies with the activity of the enzyme observed with NADPH method (similar patterns), which validates that the AlkB has been reconstituted more efficiently in RPEM than other two methods.

### Native page and zymography of reconstituted AlkB

**Figure 8:**
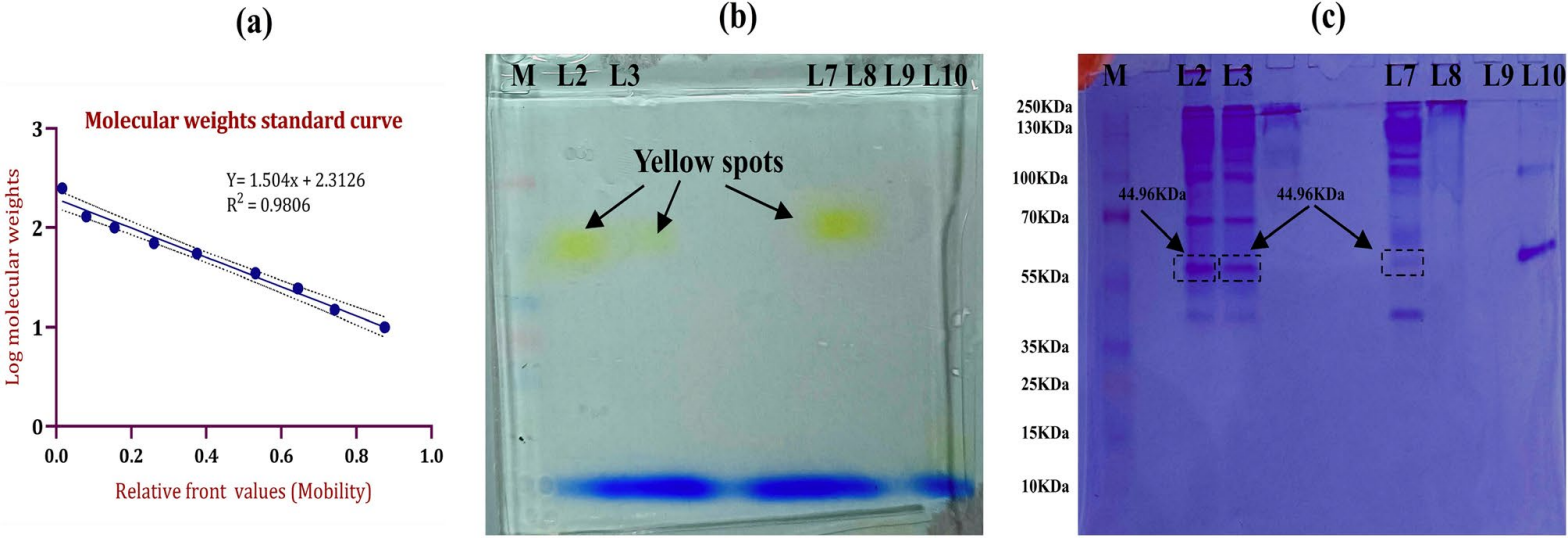
Gel image of Native PAGE & Zymography of AlkB. (**a)** is the standard plot of the log molecular weight of the marker, (**b)** is the zymography of AlkB after activity staining with 8-Pnpane, and (**c)** is the native-PAGE gel after Coomassie Brilliant Blue staining. The lanes are as follows: M: Marker; Lane 2&3: Concentrated crude samples; Lane 7: Concentrated liposome sample from RPEM; Lane 8: Concentrated liposome sample from DMB; Lane 9: Concentrated liposome sample from EIM; Lane10: BSA.

Since the Native PAGE is the only way to visualize the protein/enzyme in its native state which can be further analyzed by activity staining/zymography of the desired enzyme. In this study, AlkB is resolved on 11% polyacrylamide gel and the protein bands are very clearly visible along with the protein marker (Fig. 8). The RPEM sample shows almost similar band intensity as the crude enzyme sample does, whereas samples from other two methods are not that much intense and clear. The zymography analysis of AlkB shows the very precise yellow spot on the gel in lanes 3 & 4 (crude enzyme) and lane 7 (RPEM sample) (Fig. 8b). The molecular weight of AlkB was calculated approx 44.6 kDa after coomassie staining (Fig. 8c) with the help of standard plot of log molecular weight of protein markers (Fig. 8a) and there is no yellow spots are present in DBM and EIM samples. However, some other protein bands can be also observed except AlkB after CBB staining (Fig. 8c).

### Thermostability of AlkB in different liposomes

**Figure 9:**
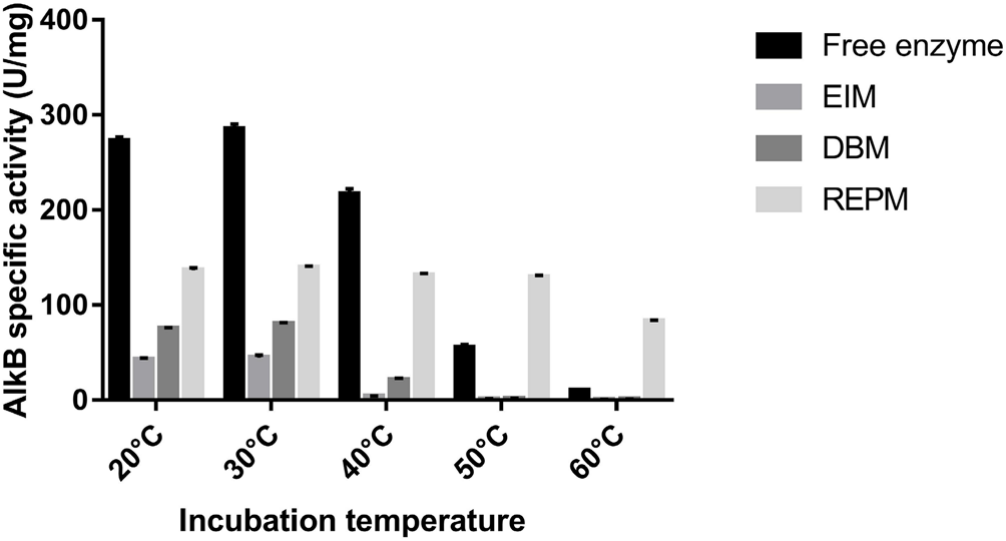
Thermal stability of reconstituted AlkB enzyme in different methods along with free enzyme. Data are shown as the mean of triplicate data after a one-way ANOVA test which indicates statistical significance (*P* = 0.0092* and n* = 3).

An effect of temperature on AlkB activity was examined up to 60 °C. The thermostability of AlkB encapsulated through EIM and DBM methods shows the decline in AlkB activity after 30 °C while with the RPEM method, it was stable up to 50 °C and then started decreasing (Fig. 9).

## Discussion

The reconstitution of membrane proteins in native lipid bilayers is a promising approach with numerous applications. It provides controlled physiochemical stability to enzymes and, a biocompatible environment for enzymes and stability. In the current study, a novel approach has been explored for partial purification of alkane hydroxylase (AlkB) by encapsulating it into a liposome, on restructuring the native lipid bilayer of *Penicillium* *chrysogenum* SNP5 to extend the native and protective environment to the enzyme. Three different methods i.e., reverse-phase evaporation method, detergent-based method, and ethanol injection method (EIM) have been used for reconstituting its native membrane into liposome.

The physicochemical characterization and assessment of reconstituted liposomes have been done in order to check the in vitro stability of encapsulated enzymes through fluorescence microscopy, AFM, particle size analysis, AlkB-specific activity, zymography, and thermostability of AlkB. The Fluorescence imaging reveals that the ethanol injection method produces irregular shapes and lesser populations which might be due to poor solubilization of membrane lipids in ethanol and reconstitution of AlkB (Fig. 2b_2_). The insufficient solubilization of membrane lipid into ethanol led to the development of lipid clumps during the injection process, which might be the possible cause of the bimodal size distribution of liposomes in EIM (Fig. 2b_2_). Another explanation could be that the spin coat process was used during slide preparation for AFM examination, in which liposomes might have fused together resulting in bimodal size distribution. Similar findings have been reported where a higher concentration of lipids resulted in the formation of diverse sizes of liposomes^42,43^. While Charcosset et al*.* 2015 and Laouini et al*.* 2011 achieved an appropriate population of liposomes with commercial phosphatidylcholine using the ethanol injection method^44,45^.

The detergent-based method has shown better results, but with uneven distribution of liposome, it appears that membrane lipids are sparingly soluble in digitonin and Triton X-100 and reconstitution of it might have also affected during the dialysis process Fig. 2c_2_. Whereas, Oberholzer et al*.* 1999 have reported the proper synthesis and reconstitution of glucose-1-phosphate using commercial phospholipids with this method^46^. With the RPEM method liposomes of better uniformity and lamellarity were synthesized due to the formulation of an appropriate solvent mixture (chloroform: methanol) to dissolve and reassemble the cell membrane which might have provided an amphipathic environment and helpful in reassembling liposomes Fig. 2d_2_. Another reason might be the composition of phospholipids of the membrane which may differ from organism to organism, might have a higher solubility in the solvents used in RPEM, and might have provided a suitable microenvironment for reconstitution.

Liposomes prepared through RPEM were high in AlkB enzyme activity, while assaying AlkB activity using the NADPH method (Fig. 5) and 8-pnpane method (Fig. 7), whereas the other two methods failed to reconstitute the AlkB in terms of activity and stability it might be due to the use of a proper solvent system (i.e., chloroform and methanol in a 2:1 ratio) for dissolution of membrane phospholipids and reconstitution. It has been reported that reverse-phase evaporation provides higher encapsulation efficiency and higher stability when used for the oxidoreductase class of enzymes or membrane-bound enzymes^47,48^. A similar finding has been reported for the synthesis of soybean lecithin liposomes for encapsulation of D-( +)-Glucose^49^ and Soy phosphatidylcholine, Phospholipon 90 (PC) for encapsulation of retinyl acetate and sodium cromoglycate drugs^50^.

The outcome from native PAGE and zymography was an interesting finding in this study. A clear yellow color band of AlkB appeared in the zymogram of the sample lane of RPEM liposomes, whereas, yellow band was absent in DBM and EIM sample lanes which support the findings of AFM. To visualize the AlkB protein in Zymogram, 8-pnpane was used as an enzyme substrate, which breaks into yellow-colored p-nitrophenolate and Fatty acid in presence of AlkB (Fig. 1). Here, 8-pnpane method is used instead of NADH method because 8-pnpane is a very specific substrate for AlkB and generates color as well and also to eliminate the confusion with other NADH dependent dehydrogenases present in cells and membrane to check its purity. This is the first report where, 8-pnpane is used to visualize AlkB in zymography and the presence of 45KDa AlkB, in *Penicillium* *chrysogenum* SNP5*,* which may open new avenues for *Penicillium* *chrysogenum* *SNP5* for industrial applications. Al-Kanany et al*.* 2020 have reported the molecular weight of AlkB as 46 KDa in *Pseudomonas* *aeruginosa* and Marín et al*.* 2001 reported 43.95KDa in *Burkholderia* *cepacia* RR10 which indicates that the AlkB of *Penicillium* *chrysogenum* *SNP5* also falls in the similar range of molecular weights^51,52^. The presence of other protein bands on Native-PAGE gel after CBB staining might be due to the reconstitution of coenzymes of AlkB like rubredoxin and rubredoxin reductase, enzymes of cascade of AlkB metabolism like alcohol dehydrogenase and aldehyde dehydrogenase.

According to the thermostability analysis of AlkB, encapsulated through the reverse-phase evaporation method has shown a stronger resistance against thermal denaturation in comparison to the free enzyme and the other two methods. The obtained results could be attributed to the proper reassembling of membrane lipids in the presence of a chloroform and methanol mixture, which increases the thermal stability of reconstituted enzymes. Similar results have been reported by Yoshimoto et al*.* 2007 for the encapsulation of catalase and by Yoshimoto et al*.* 2008 for the encapsulation of alcohol dehydrogenase^53,54^. Both studies demonstrated that reverse-phase evaporation-produced liposomes exhibit higher thermostability than other techniques.

It can be concluded that among all three different methods along with control, uniform, even distributed liposomes with high specific activity of AlkB and of appropriate size, liposomes were synthesized through the reverse-phase methodology. This was achieved by selecting a suitable solvent mixture and their ratio was optimized to achieve a high concentration of liposome with high specific activity of AlkB. Another advantage of the selected solvent mixture was that it evaporated completely upon completion of the process without denaturing the liposomes.

### Statistical analysis

All experiments were performed in triplicates and statistical analysis was done with one-way ANOVA and considered to be significant at *p* < 0.05 in this study.

## Conclusion

The present findings conclude that the reconstitution of the plasma membrane into liposomes through the reverse phase method is a very promising approach to isolating the whole cascade of integral proteins or enzymes in its native form. In the current study AlkB enzyme cascade in its functional form has been enclosed in the liposomes synthesized by reconstructing the membrane lipid of *Penicillium* *chrysogenum* through the reverse phase method successfully, that possess high thermostability and catalytic activity as well. Current findings for the isolation of AlkB in its native form along with its coenzymes would increase its applications and exploration for a wide variety of industrial applications.

## Data Availability

The datasets generated during and/or analyzed during the current study are available from the corresponding author upon reasonable request.
